# Differential control of sympathetic outflow to muscle and skin during physical and cognitive stressors

**DOI:** 10.1007/s10286-024-01015-6

**Published:** 2024-02-03

**Authors:** Brendan McCarthy, Sudipta Datta, Gianni Sesa-Ashton, Rebecca Wong, Tye Dawood, Vaughan G. Macefield

**Affiliations:** 1 Baker Heart and Diabetes Institute, Melbourne, VIC Australia; 2 Baker Department of Cardiometabolic Health, The University of Melbourne, Melbourne, VIC Australia; 3 Department of Anatomy and Physiology, The University of Melbourne, Melbourne, VIC Australia; 4 Department of Neuroscience, Central Clinical School, Monash University, 99 Commercial Road, Melbourne, VIC 3004 Australia

**Keywords:** Cognitive stress, Peristimulus time histogram, Physical stress, Sympathetic nerve activity

## Abstract

**Purpose:**

Sympathetic nerve activity towards muscle (MSNA) and skin (SSNA) regulates various physiological parameters. MSNA primarily functions in blood pressure and flow, while SSNA operates in thermoregulation. Physical and cognitive stressors have been shown to have effects on both types of sympathetic activity, but there are inconsistencies as to what these effects are. This article aims to address the discrepancies in the literature and compare MSNA and SSNA responses.

**Methods:**

Microelectrode recordings were taken from the common peroneal nerve in 29 participants: MSNA (*n* = 21), SSNA (*n* = 16) and both MSNA and SSNA (*n* = 8). Participants were subjected to four different 2-min stressors: two physical (isometric handgrip task, cold pressor test) and two cognitive (mental arithmetic task, Stroop colour–word conflict test), the latter of which saw participants separated into responders and non-responders to the stressors. It was hypothesised that the physical stressors would have a greater effect on MSNA than SSNA, while the cognitive stressors would operate conversely.

**Results:**

Peristimulus time histogram (PSTH) analysis showed the mental arithmetic task to significantly increase both MSNA and SSNA; the isometric handgrip task and cold pressor test to increase MSNA, but not SSNA; and Stroop test to have no significant effects on changing MSNA or SSNA from baseline. Additionally, stress responses did not differ between MSNA and SSNA in participants who had both sets of data recorded.

**Conclusions:**

This study has provided evidence to support the literature which claims cognitive stressors increase sympathetic activity, and provides much needed SSNA data in response to stressors.

## Introduction

Sympathetic nerve activity to muscle and skin can be recorded directly in humans via a tungsten microelectrode inserted percutaneously into an accessible peripheral nerve (microneurography). Muscle sympathetic nerve activity (MSNA) consists of spontaneous bursts of action potentials generated by unmyelinated postganglionic vasoconstrictor axons. This nerve activity contributes to the beat-to-beat control of blood pressure through its actions on total peripheral resistance [1]. Skin sympathetic nerve activity (SSNA), on the other hand, is composed of spontaneous bursts of action potentials generated by unmyelinated postganglionic sympathetic axons supplying blood vessels, sweat glands and the hairs. As such, SSNA is primarily involved in thermoregulation and emotional responses [1].

Certain stressors have long been used as methods of experimentally inducing changes in MSNA and SSNA. The physical stressors have a wealth of literature supporting their capacity to influence sympathetic nerve activity. Isometric handgrip tasks are known to induce increases in both MSNA and SSNA [2–4] and are easily reproducible methods of initiating increases in blood pressure, which proves useful in autonomic testing [5]. The cold pressor test, however, has been shown to increase MSNA whilst leaving SSNA unaltered [6, 7] and, similar to the handgrip test, is used clinically to assess autonomic and cardiovascular disorders through sympathetic vasomotor processes [8]. Cognitive stressors, on the contrary, are somewhat more ambiguous in efficacy under these conditions. For instance, the Stroop colour–word test is commonly used clinically and experimentally to evaluate one’s capacity to inhibit cognitive interference [2]. While this task does not frequently see usage in autonomic evaluation and its ability to modulate sympathetic nerve activity is contentious [5, 6], it does, however, have notable validity as a cognitive stressor—influencing sympathetic factors such as heart rate, blood pressure and sweat release [9, 10]. Moreover, mental arithmetic tasks have been shown to increase MSNA and SSNA [11, 12] as well as blood pressure, just as isometric handgrip tasks are used for autonomic testing [13]. With this being said, other studies have shown that mental arithmetic can decrease activity [14, 15] or leave it unaltered [16–20]. The capacity for MSNA and SSNA to respond differently from each other under different stress conditions is due to their respective properties. As touched on above, MSNA is tightly coupled to changes in blood pressure, while SSNA is dependent upon thermoregulatory and emotional stimuli [1]. As such, different stressors and, indeed, disease states [21] will affect MSNA and SSNA differentially according to the outcomes of the stressor.

We sought to evaluate the effects of each of the above stressors on MSNA and SSNA at four time points—before, at the beginning, at the end, and after the stressors—in order to determine the efficacy of each stressor (both physical and cognitive) on altering sympathetic activity. This stems from the inconsistencies in the literature as to the effects of the cognitive stressors, as well as the relative lack of studies analysing the effects of stressors on SSNA. These inconsistent findings can be attributed, in part, to highly variable inter-individual responses to stressors. In order to account for this, we split participants into those who showed a sympathetic response to the cognitive stressors and those who did not. Additionally, rather than relying solely on the standard metrics for reporting sympathetic nerve activity (bursts/minute and bursts/100 heart beats), we used a more sensitive measure: electrocardiogram (ECG) R-wave-triggered sympathetic spike counts. In analysing data using these peristimulus time histograms (PSTH) referenced to the cardiac cycle, the effects of heart rate are removed and MSNA and SSNA can be more fairly compared between stressors—differences in activity could be almost solely attributed to the stressors themselves [22]. In addition, our sample included subjects from which we were able to obtain both MSNA and SSNA recordings on separate occasions. PSTHs are capable of facilitating a comparison of MSNA and SSNA *within* participants during stressors—a parameter that, to the best of our knowledge, has not been evaluated as of yet. With these aims in mind, we hypothesised that the physical stressors would have more effects on MSNA than SSNA, while the cognitive stressors would operate in the opposite manner, given the known properties of these stressors.

## Methods

### Subjects and ethics

Thirty-seven sets of data were obtained (21 MSNA; 16 SSNA) from 29 healthy volunteers—eight volunteers elected to participate again in order to obtain both MSNA and SSNA recordings. In the case of repeated participants, an interval of approximately 1–4 months elapsed between visits. The generally accepted practice is to wait at least 4 weeks between sessions when recording from the same nerve in order to reduce the risk of nerve inflammation [1]. This practice is of little consequence as it has been shown that intra-individual sympathetic nerve activity is highly stable over the time course of years [23]. MSNA data comprised recordings taken from 11 male and 10 female participants (age 21–40 years, mean ± standard deviation (SD) 25.4 ± 5.6 years; height 155–193 cm, 170.3 ± 10.9 cm; weight 50–91 kg, 66.3 ± 11.1 kg), while SSNA data comprised recordings taken from 10 male and six female participants (age 20–35 years, 24.3 ± 4.8 years; height 155–186 cm, 170.3 ± 8.8 cm; weight 52–81 kg, 67.7 ± 9.5 kg). Each data set was obtained specifically for this study and, as such, has not been previously reported. The study was approved by the Human Research Ethics Committee of the Alfred Hospital (HREC approval 467/19), endorsed by Governance of the Baker Heart and Diabetes Institute and conformed to the Declaration of Helsinki, with all participants providing informed written consent. Each participant was instructed to refrain from digestion of caffeinated food and beverages and the intake of nicotine on the morning of the experiment, as these are known to influence sympathetic activity [21, 24].

### Recording procedures

Microneurography was conducted on all participants in order to obtain MSNA or SSNA recordings via the right (*n* = 36) or left (*n* = 2) common peroneal nerve around the fibular head. For a detailed description of the microneurography methods, we refer the reader to Sesa-Ashton et al*.* [25] for MSNA and Wong et al. [26] for SSNA. As described in detail previously [1], while intraneural electrical stimulation identified the fascicle as supplying muscle or skin, this was confirmed by testing the responses to muscle stretch or tendon percussion as well as light stroking over the skin of the innervation territory. Sustained increases in spontaneous cardiac-locked bursts of MSNA but not SSNA during a maximal inspiratory capacity apnoea, and an arousal burst of SSNA but not MSNA to an unexpected loud auditory stimulus, were used to differentiate between the two types of sympathetic outflow. In addition to microneurography, continuous ECG recordings were sampled at 2 kHz via three 35-mm Ag/AgCl surface electrodes (Covidien, Ireland) placed on the chest and continuous non-invasive blood pressure recordings were obtained from two finger cuffs, sampled at 400 Hz and calibrated to a sphygmomanometer cuff on the contralateral arm (NOVA, Finapres Medical System BV, the Netherlands).

### Stress procedures

Participants were subjected to four different stress conditions: an isometric handgrip task in which subjects grasped a grip force transducer (ADInstruments, Australia) at 30–35% of their maximal voluntary contraction (MVC) force (determined prior to the beginning of the task); a cold pressor task in which subjects submerged their dominant hand into a bucket of ice water; a mental arithmetic task in which participants were instructed to perform continuous arithmetical subtraction of seven from a randomly chosen three-digit number whilst being subjected to negative verbal treatment in a loud and distracting environment; and a Stroop colour–word conflict test task. Each stressor lasted for 2 min, with the exception of the Stroop test which participants were instructed to finish as fast as possible. Upon conclusion of each stress task, participants were instructed to report their perceived level of stress on a scale of 0 to 10, with 0 being not at all stressful and 10 being the worst stress imaginable. Technical difficulties such as poor signal-to-noise ratio and loss of recording site due to leg movement prevented some participants from undertaking every stressor. This resulted in sample sizes of 21 MSNA and 16 SSNA for handgrip, 18 MSNA and 14 SSNA for cold pressor, 16 MSNA and 12 SSNA for mental arithmetic, and 20 MSNA and 16 SSNA for the Stroop test. Stressors were delivered in randomised order and an approximately 5-min rest interval was provided between stressors.

### Data analysis

In order to ensure that the data were not misrepresented by positive-going spikes from myelinated axons or single motor units [27], the primary analysis was conducted on the raw, negative-going MSNA and SSNA spikes. These negative-going spikes (defined with a half width of 0.2–0.5 ms), as well as R-waves of the ECG were detected using window discriminator software on LabChart 7 (Spike Histogram for Macintosh, v2.5.1, ADInstruments, Australia). The same software was also used to generate autocorrelation histograms of the nerve activity and cross-correlation histograms of activity referenced to the cardiac cycle. In each, an average burst of activity was represented and displayed with a peak at time 0, with prior and future average activity displayed in negative and positive time values, respectively.

Furthermore, PSTHs were generated from which the primary results were obtained. The average burst referenced to the R-waves of the ECG across the entire data file was first determined in each participant and used as a primer for the duration of bursts in each subsequent analysis—that being a selection of 50 R-waves taken from time points immediately before, at the start, at the end and after each stressor. The corresponding nerve activity during each of these times was computed from those bins of the histogram encompassing the start and end of the average sympathetic burst and recorded as spikes per 50 R-waves.

These spikes were then compared within each stressor through a statistical and graphical analysis program (Prism 9 for Macintosh, v9.1.2, GraphPad Software, USA). D’Agostino and Pearson normality tests were utilised to ascertain normality—a repeated measures one-way analysis of variance (RM one-way ANOVA) followed by a Tukey’s multiple comparisons test or a Friedman test followed by a Dunn’s multiple comparisons test were used for data which passed or failed normality, respectively. Further analysis of the cognitive stressors required data to be separated into groups of responders and non-responders. This was chosen on the basis of PSTH sympathetic spike count at the last 50 R-wave time period ≥ 10% or < 10% increased from the baseline period, respectively. Given the reduced sample size, responder data were tested for normality using a Shapiro–Wilk normality test, before undergoing further analysis as described above. Additionally, data were compared at the same time points relative to the stressors within the eight participants who yielded both MSNA and SSNA recordings. Here, a mixed-effects analysis with a Šidák’s multiple comparisons test was used to analyse and compare grouped data.

Standard measures of multi-unit sympathetic nerve activity—burst frequency (bursts/minute), burst incidence (bursts/100 heart beats) and total burst activity (cumulative burst amplitude in 1 min)—were obtained at rest and during each manoeuvre from the root-mean square (RMS)-processed nerve signal (200 ms moving average). Data were split into time periods of 2 min before and after each stressor as well as into the first and second half of each stressor. Heart rate and systolic and diastolic blood pressure were analysed in the same time periods. Technical issues prevented the recording of blood pressure during the handgrip and cold pressor tasks for one participant in the SSNA data set, reducing the sample for those tasks by one. All these data were tested for normality and significance in the same manner as the PSTH data described above. In all analyses, data are presented as mean ± SD; differences were considered significant if they attained a* P* value of less than 0.05.

## Results

Successful recordings of sympathetic nerve activity were taken from 29 participants, eight of whom were studied on a second occasion (to record SSNA if MSNA was recorded in the first session, and vice versa), resulting in 37 data sets: 21 MSNA and 16 SSNA. Raw, negative-going sympathetic spikes were analysed using PSTHs referenced to time blocks of 50 R-waves. Each participant was subjected to at least two of the four following stressors (technical difficulties prevented all four stressors from being conducted in some experiments): a handgrip task, a cold pressor test, a mental arithmetic task and a Stroop colour–word conflict challenge. Standard metrics of MSNA and SSNA—burst frequency, burst incidence and total burst activity at rest and during the manoeuvres—are provided in Tables 1 and 2, along with significant changes from baseline. Values for the standard metrics were taken from a 2-min period before and after each stressor as well as during the first half and second half of each stressor.

**Table 1:** RMS-processed MSNA metrics during each stressor

		Handgrip	Cold pressor	Mental arithmetic	Stroop test
Burst frequency (bursts/min)	Baseline	21.2 ± 5.0	21.4 ± 5.4	21.2 ± 3.6	20.3 ± 4.5
	First half of stressor	21.0 ± 5.6	22.7 ± 6.4	18.8 ± 4.4*	19.2 ± 4.6
	Second half of stressor	23.1 ± 6.5	23.2 ± 9.9	20.1 ± 3.6	19.2 ± 4.8
	Recovery	21.7 ± 5.0	23.4 ± 7.0	22.6 ± 4.7	21.1 ± 4.1
Burst incidence (bursts/100 heart beats)	Baseline	31.1 ± 7.7	31.2 ± 7.6	31.7 ± 5.6	30.4 ± 7.9
	First half of stressor	28.3 ± 8.3	29.6 ± 8.7	23.4 ± 5.5****	27.0 ± 6.8
	Second half of stressor	29.6 ± 9.5	35.3 ± 14.7	26.3 ± 5.2	26.8 ± 7.6
	Recovery	31.3 ± 8.6	35.8 ± 11.8	31.6 ± 5.4	31.0 ± 6.8
Cumulative burst amplitude (µV)	Baseline	10.1 ± 6.8	10.1 ± 8.0	8.1 ± 7.1	10.9 ± 7.2
	First half of stressor	10.9 ± 7.5	14.8 ± 13.3	10.8 ± 7.6	8.9 ± 7.9*
	Second half of stressor	15.4 ± 10.7**	17.9 ± 16.0***	11.7 ± 7.2*	8.9 ± 7.8
	Recovery	10.9 ± 6.9	13.3 ± 10.6	10.6 ± 7.9*	9.9 ± 7.2

The standard metrics for MSNA—burst frequency (taken as bursts per minute), burst incidence (taken as bursts per 100 heart beats) and cumulative burst amplitude—are displayed during a time period before, during the first half, during the second half and after each stressor. Time points which were found to be significantly different from the baseline during each stressor are denoted with an asterisk (*P < 0.05; **P < 0.01; ***P < 0.001; ****P < 0.0001). Data are presented as mean ± SD

**Table 2:** RMS-processed SSNA metrics during each stressor

		Handgrip	Cold pressor	Mental arithmetic	Stroop test
Burst frequency (bursts/min)	Baseline	10.6 ± 9.3	12.1 ± 11.3	11.5 ± 10.1	10.8 ± 8.8
	First half of stressor	16.3 ± 11.7	17.1 ± 13.0	14.8 ± 12.5	12.6 ± 10.6
	Second half of stressor	20.9 ± 15.2*	9.2 ± 10.1	14.7 ± 14.5	12.0 ± 12.9
	Recovery	8.9 ± 8.1	10.0 ± 9.4	11.2 ± 12.2	10.3 ± 10.3
Burst incidence (bursts/100 heart beats)	Baseline	14.0 ± 11.3	15.7 ± 14.6	14.5 ± 9.9	14.2 ± 11.4
	First half of stressor	19.6 ± 13.1	19.3 ± 14.5	18.1 ± 15.2	16.8 ± 13.2
	Second half of stressor	22.7 ± 13.7	11.6 ± 13.6	18.2 ± 17.8	15.7 ± 15.9
	Recovery	11.9 ± 10.2	13.1 ± 11.6	13.8 ± 13.6	14.3 ± 14.3
Cumulative burst amplitude (µV)	Baseline	4.9 ± 5.1	6.0 ± 7.6	4.7 ± 4.5	5.1 ± 5.6
	First half of stressor	8.1 ± 7.2	8.6 ± 8.3	6.8 ± 6.4	6.2 ± 7.0
	Second half of stressor	10.4 ± 8.4**	5.1 ± 7.8	6.1 ± 6.7	5.8 ± 8.3
	Recovery	4.7 ± 5.3	4.4 ± 4.6	4.9 ± 5.4	4.8 ± 7.1

The standard metrics for SSNA—burst frequency (taken as bursts per minute), burst incidence (taken as bursts per 100 heart beats) and cumulative burst amplitude—are displayed during a time period before, during the first half, during the second half and after each stressor. Time points which were found to be significantly different from the baseline during each stressor are denoted with an asterisk (*P < 0.05; **P < 0.01). Data are presented as mean ± SD

Furthermore, hemodynamic data—heart rate, systolic and diastolic blood pressures at rest and during the manoeuvres—are provided in Tables 3 and 4. Data were generated from the same time points as the standard metrics and significant changes from baseline are noted.

**Table 3:** Hemodynamic data from MSNA recordings during each stressor

		Handgrip	Cold pressor	Mental arithmetic	Stroop test
Heart rate (beats/min)	Baseline	68.8 ± 8.2	69.1 ± 8.6	67.8 ± 10.2	68.1 ± 7.4
	First half of stressor	75.6 ± 10.5***	77.6 ± 7.6***	81.2 ± 10.5****	71.7 ± 7.6
	Second half of stressor	80.4 ± 12.5****	74.0 ± 8.0	77.2 ± 9.4**	72.4 ± 7.8**
	Recovery	68.5 ± 8.6	66.8 ± 9.7	69.0 ± 9.7	69.2 ± 7.8
Systolic blood pressure (mmHg)	Baseline	124.1 ± 15.6	127.2 ± 26.7	127.1 ± 12.0	125.2 ± 13.0
	First half of stressor	135.3 ± 14.5****	141.2 ± 26.9*	138.5 ± 14.5***	132.2 ± 12.9***
	Second half of stressor	146.0 ± 14.8****	152.5 ± 28.9****	140.0 ± 15.3*	134.5 ± 12.7***
	Recovery	128.5 ± 10.9	130.5 ± 10.8	126.4 ± 12.1	126.4 ± 11.7
Diastolic blood pressure (mmHg)	Baseline	72.3 ± 9.6	76.4 ± 16.4	73.8 ± 6.9	75.2 ± 6.6
	First half of stressor	80.4 ± 8.3***	89.5 ± 15.5***	82.6 ± 7.7**	78.3 ± 7.3****
	Second half of stressor	91.1 ± 10.2****	96.3 ± 18.2****	86.8 ± 8.3****	79.8 ± 7.6****
	Recovery	75.1 ± 8.5	79.2 ± 8.2	76.7 ± 7.9	75.0 ± 7.8

Heart rate, systolic blood pressure and diastolic blood pressure were calculated before, during the first half, during the second half and after each stressor. Time points that were significantly different from the baseline during each stressor are denoted with an asterisk (*P < 0.05; **P < 0.01; ***P < 0.001; ****P < 0.0001). Data are presented as mean ± SD

**Table 4:** Hemodynamic data from SSNA recordings during each stressor

		Handgrip	Cold pressor	Mental arithmetic	Stroop test
Heart rate (beats/min)	Baseline	72.8 ± 13.4	73.0 ± 12.4	73.7 ± 12.8	72.1 ± 13.1
	First half of stressor	81.1 ± 17.2**	88.1 ± 16.6***	86.8 ± 19.4***	74.1 ± 14.6
	Second half of stressor	86.1 ± 15.6****	82.2 ± 13.1	85.4 ± 18.3****	74.2 ± 13.2
	Recovery	71.8 ± 13.2	71.7 ± 13.6	75.1 ± 15.2	72.4 ± 11.7
Systolic blood pressure (mmHg)	Baseline	116.2 ± 11.3	123.6 ± 14.4	116.1 ± 23.0	128.4 ± 14.6
	First half of stressor	131.7 ± 18.6**	138.2 ± 12.9	132.0 ± 12.7	134.3 ± 12.8*
	Second half of stressor	144.7 ± 17.6****	154.4 ± 18.3****	136.4 ± 11.5***	130.8 ± 13.1
	Recovery	125.3 ± 11.8*	134.7 ± 10.3	127.2 ± 10.4	124.6 ± 9.8
Diastolic blood pressure (mmHg)	Baseline	69.2 ± 10.6	70.6 ± 11.2	75.6 ± 7.6	77.7 ± 5.9
	First half of stressor	79.2 ± 17.8**	86.4 ± 10.3***	82.3 ± 6.3*	80.6 ± 5.4
	Second half of stressor	90.5 ± 12.5****	95.9 ± 9.8****	85.5 ± 6.4***	80.4 ± 6.0
	Recovery	72.0 ± 6.7	78.8 ± 4.7	76.3 ± 5.4	75.7 ± 5.2

Heart rate, systolic blood pressure and diastolic blood pressure were calculated before, during the first half, during the second half and after each stressor. Time points which were found to be significantly different from the baseline during each stressor are denoted with an asterisk (*P < 0.05; **P < 0.01; ***P < 0.001; ****P < 0.0001). Data are presented as mean ± SD. Technical issues required the removal of one data set from the systolic and diastolic blood pressure data during the handgrip and cold pressor tasks

### PSTH analysis of MSNA

**Fig. 1:**
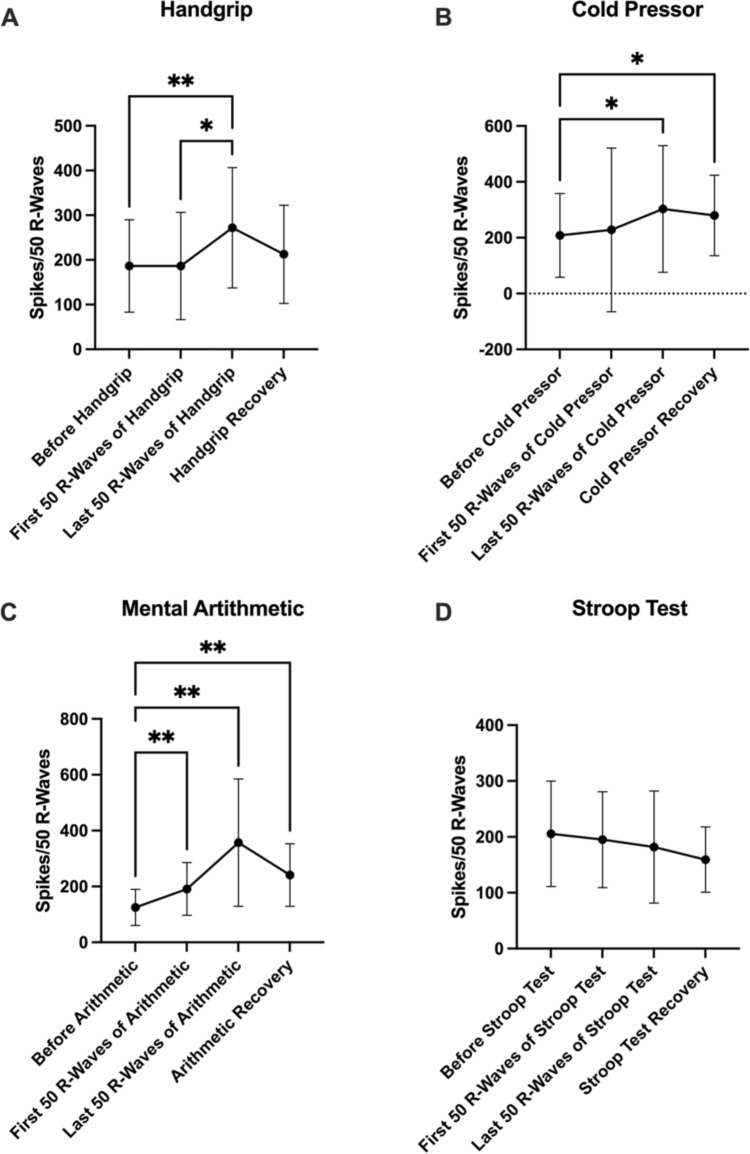
MSNA spikes during each stressor. MSNA spikes, as revealed through PSTH analysis, are shown for 50 R-wave time periods before, at the start, at the end and after **a** the handgrip task (*n* = 21), **b** the cold pressor test (*n* = 18), **c** the mental arithmetic task (*n* = 16) and **d** the Stroop colour–word test conflict (*n* = 20). Significant differences between time periods are indicated with an asterisk (*P < 0.05; **P < 0.01). Results are presented as mean ± SD

Peristimulus time histogram (PSTH) analysis of MSNA during and around the handgrip task (*n* = 21) showed significant differences across time points. When compared to the last 50 R-waves of the stressor, MSNA spikes for both the baseline period (*P* = 0.0092) and the first 50 R-waves of the stressor (*P* = 0.0138) were found to be significantly lower in count (Table 5; Fig. 1a). Similarly, MSNA during the last 50 R-waves of the stressor was significantly higher than baseline during the cold pressor test (*P* = 0.0488; *n* = 18). The two stressors differed, however, in that the recovery period for the cold pressor test was also found to be significantly higher in activity than baseline (*P* = 0.0221) (Table 5; Fig. 1b).

**Table 5:** MSNA spikes during each stressor

	Handgrip	Cold pressor	Mental arithmetic	Stroop test
Baseline	212.4 ± 179.9	163.0 ± 113.2	153.5 ± 59.9	166.0 ± 100.2
First 50 R-waves of stressor	177.9 ± 110.4	177.1 ± 104.9	253.2 ± 157.8**	210.6 ± 106.6
Last 50 R-waves of stressor	217.4 ± 137.7**	224.4 ± 163.3*	365.6 ± 253.1**	175.2 ± 119.6
Recovery	118.4 ± 73.1	172.7 ± 137.9*	225.2 ± 166.0**	122.8 ± 77.8

The average number of MSNA spikes, as revealed through PSTH analysis, during a 50 R-wave time period before, at the start, at the end and after each stressor is displayed. Time points which were found to be significantly different from the baseline during each stressor are denoted with an asterisk (*P < 0.05; **P < 0.01). Data are presented as mean ± SD

An increase in R-wave-triggered spikes was found during the mental arithmetic task (*n* = 16) when comparing all three time points to baseline: the first 50 R-waves of the stressor (*P* = 0.0099); the last 50 R-waves of the stressor (*P* = 0.0059); and the recovery period (*P* = 0.0096) (Table 5; Fig. 1c). On the contrary, unlike the aforementioned stressors, analysis of the Stroop test (*n* = 20) revealed no significant differences between any time points (*P* > 0.05) (Table 5; Fig. 1d).

### Standard metrics analysis of MSNA

MSNA burst frequency (taken as bursts/minute; Table 1) saw no significant differences between any of the time points during the handgrip, cold pressor and Stroop tasks (*P* > 0.05). However, during mental arithmetic, the first half of the stressor was found to have a significantly lower burst frequency than both the baseline (*P* = 0.0370) and recovery (*P* = 0.0013) periods.

In a similar vein to the above, MSNA burst incidence (taken as bursts/100 heart beats; Table 1) saw no significant difference between any time points during the cold pressor and Stroop tests (*P* > 0.05). Unlike burst frequency, though, the handgrip task revealed that the first half of the stressor had significantly less burst incidence than the recovery period (*P* = 0.0430). Significant differences were also found during the mental arithmetic task, with the first half of the stressor significantly lower than the baseline (*P* < 0.0001) and recovery (*P* < 0.0001) periods. The same was true for the second half of the stressor—incidence was significantly lower than the baseline (*P* = 0.0115) and recovery (*P* = 0.0270) periods.

Each of the stressors saw significant differences in cumulative burst amplitude (Table 1). During the handgrip task, the second half of the stressor was significantly higher in cumulative burst amplitude than all the other time points: the baseline (*P* = 0.0038), first half of the stressor (*P* = 0.0019) and recovery (*P* = 0.0049) periods. The second half of the stressor was also significantly higher than the baseline (*P* = 0.0004) and recovery (*P* = 0.0402) periods during the cold pressor test. During mental arithmetic, the baseline period had significantly lower cumulative burst amplitude than both the second half of the stressor (*P* = 0.0217) and the recovery (*P* = 0.0134) period. Finally, the Stroop test had a significantly lower value during the first half of the stressor when compared to baseline (*P* = 0.0291).

### PSTH analysis of SSNA

**Fig. 2:**
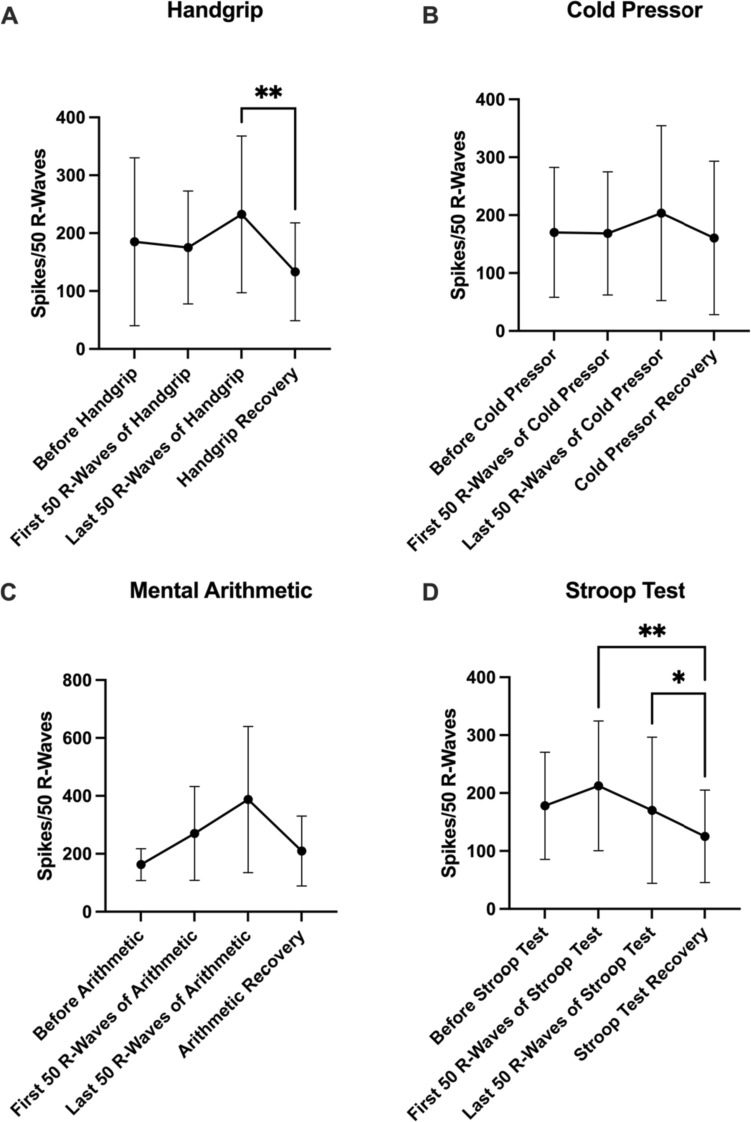
SSNA spikes during each stressor. SSNA spikes, as revealed through PSTH analysis, are shown for 50 R-wave time periods before, at the start, at the end and after **a** the handgrip task (*n* = 16), **b** the cold pressor test (*n* = 14), **c** the mental arithmetic task (*n* = 12) and **d** the Stroop colour–word conflict test (*n* = 16). Significant differences between time periods are indicated with an asterisk (*P < 0.05; **P < 0.01). Results are presented as mean ± SD

R-wave-triggered spike analysis of SSNA during the static handgrip exercise (*n* = 16) showed that the recovery period was significantly lower in SSNA than the last 50 R-wave period of the stressor (*P* = 0.0022) (Table 6; Fig. 2a). Unlike MSNA during the cold pressor test (*n* = 14), SSNA failed to reach statistical significance in comparisons between time points (*P* > 0.05) (Table 6; Fig. 2b).

**Table 6:** SSNA spikes during each stressor

	Handgrip	Cold pressor	Mental arithmetic	Stroop test
Baseline	185.2 ± 144.9	170.3 ± 112.1	162.7 ± 54.9	178.1 ± 92.6
First 50 R-waves of stressor	175.3 ± 97.5	168.4 ± 106.3	270.3 ± 162.2	212.5 ± 111.9
Last 50 R-waves of stressor	232.6 ± 135.3	203.6 ± 151.0	387.3 ± 252.6	170.3 ± 126.2
Recovery	133.3 ± 84.5	160.6 ± 132.6	209.7 ± 120.6	125.3 ± 79.9

The average number of SSNA spikes, as revealed through PSTH analysis, during a 50 R-wave time period before, at the start, at the end and after each stressor is displayed. No time points during each stressor were found to be significantly different from their corresponding baseline values. Data are presented as mean ± SD

Similar to the above, SSNA multiple-comparisons analysis did not reveal any statistically significant differences between time points (*P* > 0.05) during mental arithmetic (*n* = 12) (Table 6; Fig. 2c). Finally, the total number of spikes during the recovery period of the Stroop test (*n* = 16) was significantly lower than the first 50 R-waves of the stressor (*P* = 0.0011) and the last 50 R-waves of the stressor (*P* = 0.0405) (Table 6; Fig. 2d).

### Standard metrics analysis of SSNA

In somewhat of an inverse manner as seen with MSNA burst frequency, SSNA burst frequency (Table 2) was not found to have significant differences during the mental arithmetic and Stroop tasks (*P* > 0.05). Significant differences were found, however, during the handgrip task in which the second half of the stressor was significantly higher in frequency than the baseline (*P* = 0.0300) and recovery (*P* = 0.0029) periods. The first half of the stressor was also significantly higher in frequency than the recovery period (*P* = 0.0455). Additionally, the cold pressor test saw significantly increased burst frequency during the first half of the stressor when compared to both the second half (*P* = 0.0205) and the recovery (*P* = 0.0020) periods.

Similar results to the SSNA burst frequency were seen in SSNA burst incidence (Table 2). No significant differences were found during the mental arithmetic or Stoop tasks (*P* > 0.05), but there were differences during the handgrip and cold pressor tasks. During handgrip, the recovery period was significantly lower in burst incidence than the first half of the stressor (*P* = 0.0455), as well as the second half of the stressor (*P* = 0.0124). During the cold pressor task, the recovery period was only significantly lower in burst incidence than the first half of the stressor (*P* = 0.0325).

Cumulative burst amplitude analysis of SSNA (Table 2) saw more or less the same results as burst frequency and incidence. No significant differences were found during the mental arithmetic task (*P* > 0.05), but this time a difference was seen during the Stroop task: the recovery period was significantly lower in amplitude than the first half of the stressor (*P* = 0.0370). As with burst frequency, cumulative burst amplitude saw the second half of the stressor significantly increased from both the baseline (*P* = 0.0077) and recovery (*P* = 0.0029) periods during the handgrip task. Also similar to burst frequency, cumulative burst amplitude during the cold pressor task saw the first half of the stressor significantly higher in value than the second half of the stressor (*P* = 0.0259) and the recovery period (*P* = 0.0015).

### Cognitive stressor responders

As it is known that some individuals have a negative sympathetic response to cognitive stressors, subjects were separated into responders and non-responders. MSNA mental arithmetic (*n* = 13) saw similar results to the pooled data in that baseline was significantly different to each other time point (first 50 R-waves, *P* = 0.0208; last 50 R-waves, *P* = 0.0034; recovery, *P* = 0.0034) (Table 7). A different result was found, however, in that the last 50 R-wave time period was now significantly greater in spike count than the first 50 R-wave time period (*P* = 0.0373), too (Table 7). Once the responders (*n* = 6) were identified for the MSNA Stroop test, a significant difference between the last 50 R-waves time point and the recovery period (*P* = 0.0046) was found as well (Table 7). SSNA mental arithmetic (*n* = 9) saw an increase at the last 50 R-wave time period from baseline (*P* = 0.0061) (Table 7). The same significant comparisons were found for the SSNA Stroop test as the pooled data, once responders (*n* = 6) had been split from non-responders (Table 7), but here, again, the level of significance was changed (*P* = 0.0478 for the last 50 R-waves vs recovery and no significant difference (*P* > 0.05) between the first 50 R-waves and recovery).

**Table 7:** Sympathetic activity of responders during cognitive stressors

	MSNA		SSNA	
	Mental arithmetic	Stroop test	Mental arithmetic	Stroop test
Baseline	121.1 ± 63.8	151.7 ± 82.7	148.3 ± 55.4	156.8 ± 108.5
First 50 R-waves of stressor	193.7 ± 93.2*	196.7 ± 77.5	271.3 ± 174.2	264.8 ± 139.3
Last 50 R-waves of stressor	405.6 ± 223.9**	240.0 ± 73.3	458.6 ± 254.5**	270.8 ± 152.1
Recovery	265.6 ± 101.6**	170.5 ± 51.7	237.7 ± 128.3	178.2 ± 108.3

The average number of MSNA and SSNA spikes, as revealed through PSTH analysis, during a 50 R-wave time period before, at the start, at the end and after each cognitive stressor is displayed for participants who showed a ≥ 10% increase in sympathetic activity from baseline by the last 50 R-waves time point. Time points which were found to be significantly different from the baseline during each stressor are denoted with an asterisk (*P < 0.05; **P < 0.01). Data are presented as mean ± SD

The same participants identified as responders during PSTH analysis were analysed using the standard metrics as well. The MSNA mental arithmetic task saw the same results as the grouped cohort in each of the metrics. The first half of the stressor had lower burst frequency than the baseline (*P* = 0.0022) and recovery (*P* = 0.0107) periods, as well as lower burst incidence than the baseline (*P* < 0.0001) and recovery (*P* < 0.0001) periods. Cumulative burst amplitude analysis also revealed the baseline period to be significantly lower than the second half of the stressor (*P* = 0.0002) and the recovery (*P* = 0.0050) period. Different results were seen in regards to the MSNA Stroop test, however. Here, burst frequency analysis showed the second half of the stressor to be significantly lower in activity than the recovery period (*P* = 0.0476). Burst incidence analysis revealed the first half of the stressor to be significantly lower in activity than the recovery period (*P* = 0.0268). Finally, cumulative burst amplitude analysis showed no significant differences across time points (*P* > 0.05). Also similar to the pooled responders and non-responders data, SSNA analysis of the responders during the cognitive stressors revealed no significant differences (*P* > 0.05), even in the Stroop test which yielded one significantly different result before.

### Muscle vs skin sympathetic nerve activity within participants

Having obtained both MSNA and SSNA data from eight participants on different days, analysis comparing the two types of sympathetic activity was performed during each stressor. In comparing mean activity at each time point during the handgrip (*n* = 8), cold pressor (*n* = 6), mental arithmetic (*n* = 4) and Stroop test (*n* = 8) tasks, no significant differences were found (*P* > 0.05). Similarly, once data had been normalised to baseline in order to obtain a percentage change at each time point, no significant differences were found (*P* > 0.05).

## Discussion

While the majority of studies that have quantified sympathetic nerve activity to muscle and skin have analysed the standard metrics for multi-unit activity, obtained from the RMS-processed or integrated neurogram, we have used a more sensitive measure that simply counts the number of sympathetic spikes in an R-wave-triggered histogram—a form of PSTH. This approach facilitates the comparison of MSNA and SSNA data at the inter-individual level by eliminating heart rate as a variable. Furthermore, as PSTH analysis uses raw sympathetic spikes, baseline shifts in the RMS signal associated with electromyographic noise or muscle spindle activity are mitigated. Total spikes generated during blocks of 50 R-waves were computed at baseline (immediately before the onset of the manoeuvre), during 50 R-waves at the start of the manoeuvre, during the final 50 R-waves of the manoeuvre, and during the first 50 R-waves of recovery.

### Effects of stressors on MSNA

Our isometric handgrip and cold pressor tasks increased MSNA from baseline to a statistically significant extent by the last 50 R-wave time period. This was not an unexpected result, as a wealth of literature exists verifying the efficacy of these tasks in positively modulating MSNA [4, 23, 28–30] and our own cumulative burst amplitude analysis corroborated these results. Moreover, the cold pressor task saw a sustained increase from baseline during the 50 R-wave recovery period, which was not reflected in the handgrip task. As such, these results suggest that the isometric handgrip task is capable of producing a robust, but more quickly resolving, increase in MSNA—the immediate relaxation reflecting the immediate cessation of central command. The increase in MSNA seen during the cold pressor test developed slowly but continued into the immediate recovery period. These PSTH results are incongruent with the results obtained from the standard metrics (Table 1), however. The cold pressor test did not see an increase in burst frequency or incidence, which is somewhat unexpected given the widely cited effects of this stressor on MSNA [23, 30]. One possible explanation for this comes back to the properties of PSTH analysis and the nature of the cold pressor test. During this stressor, participants had a tendency to tense up as a result of pain and discomfort—often leading to extraneous signals in the microneurographic recording. This inevitably led to messier data for use in standard metric reporting, while PSTH analysis filtered out this signal disturbance and, hence, led to results more in line with the current literature.

Mental arithmetic, just as with the isometric handgrip and cold pressor tasks, increased MSNA from baseline to a statistically significant extent by the last 50 R-wave time period, but was the only stressor to produce a significant increase in spike count within the first 50 R-waves, indicating a more rapid influence of this stressor on sympathetic outflow to the muscle vascular bed. The efficacy of the mental arithmetic task was further exemplified when the data was separated into responder and non-responder groups, given the evidence that mental stressors can induce positive or, indeed, negative influences on MSNA on an individual basis [31, 32]. An additional difference was identified between the two time points during the stressor. Results such as these are supported by Wang et al.’s study [33] in which they reported that various cardiovascular responses had rapid increases during the first 150 s of mental arithmetic before plateauing. As our own mental arithmetic protocol was conducted for only 120 s, it is reasonable to assume subjects were still within the rapid increase period. While the consistency of the task at increasing MSNA is contentious, our results and experimental design differ from two of the more commonly cited papers displaying its inhibitory properties. Firstly, Matsukawa et al. [15] used the tibial nerve at the popliteal fossa for their microneurographic recordings. As they themselves state, another group [11] demonstrated discrepancies in levels of MSNA during mental arithmetic based on the recorded nerve. Furthermore, Delius et al. [14] had a sample size of only four for their mental arithmetic task and did not account for what we now know to be inter-individual variability in cognitive stressor response (i.e. responders and non-responders). Other studies which suggest that mental arithmetic does not increase MSNA [16–20] also do not separate responders and non-responders, resulting in an averaged response in which the increase in MSNA in the responders and decrease in the non-responders essentially cancel each other out. With this being said, our own data on the standard metrics (i.e. burst frequency, burst incidence and cumulative burst amplitude) aligns with these studies—that is, our mental arithmetic protocol saw a decrease in MSNA from baseline. As such, sympathoinhibition associated with mental arithmetic is no doubt real. Additionally, we identified a lack of a response during the Stroop colour–word conflict test in the PSTH pooled data group. Thus, participants were subsequently separated into responders and non-responders. Through this analysis it was found that the last 50 R-wave time period significantly differed from the recovery period, but still no significant changes were found from baseline. In a possible explanation for this, Callister et al. [34] proposed that MSNA fluctuations during mental stress tests are directly correlated to the perceived level of stress during the test. As our MSNA participants reported a relatively low average stress rating of 3.1 ± 1.7 arbitrary units out of 10 (obtained from 20 of the 21 participants), this would appear to be expected. The six responders had a similar stress rating of 3.2 ± 1.7. However, the accuracy of the conclusion offered by Callister et al. [34] is yet to be further proven, with studies such as the one produced by Carter et al. [16] contradicting the original hypothesis.

### Effects of stressors on SSNA

The results obtained from the SSNA data set are somewhat less supported by the current body of literature, though SSNA has been far less studied than MSNA. The cold pressor test did not induce any significant increase in spike counts, as one might expect from prior work [6]. Moreover, there were no differences in direct comparison of their efficacy in altering SSNA from baseline. More surprisingly, however, handgrip also did not increase SSNA from baseline to a significant extent. This result is incongruent with prior works showing isometric handgrip tasks to have profound effects on SSNA [28, 35, 36]. Each of these studies used a relatively similar handgrip and nerve recording protocol to our own—the primary discrepancy lies in the method chosen to analyse SSNA data: using the integrated nerve signal or using the PSTH approach we used herein. Analysis conducted on the cold pressor and handgrip manoeuvres using the RMS-processed nerve signal, however, did indicate increases in SSNA during the stressors when compared to their baseline and recovery periods.

The lack of significant differences from baseline found in the physical stressors using PSTH analysis also carried over to the cognitive stressors. As SSNA is tied to an individual’s emotions [37, 38], this may be expected of the Stroop test (with the low average stress rating of 3.5 ± 2.0 arbitrary units out of 10), but mental arithmetic is known to have profound effects on SSNA in both healthy and diseased states [12, 39, 40]. Once the cognitive stressors had been separated into responders and non-responders, though, mental arithmetic showed an increase in SSNA by the last 50 R-wave time period. Therefore, it can be concluded that the non-responders dampened the results in the pooled data, leading to the lack of a significant increase. Granted, Muller et al. [12] identified increases in SSNA immediately upon commencement of the stressor, but given the lack of reports on mental arithmetic and SSNA of healthy controls with respect to time, further investigation is needed to corroborate these results. Furthermore, the Stroop test still did not reveal significant changes from baseline post-data separation. What it did reveal was a change in SSNA between the last 50 R-waves of the stressor and the recovery period. Alongside the fact that the first 50 R-waves of the stressor no longer had a statistically significant difference with the recovery period, it may be likely that the small sample size of six diminished the power of the results. This would therefore lead to the lack of significant differences between baseline and the stress condition. The one difference found between the stressor and non-stressor time points is consistent with what one would expect from SSNA (i.e. stress increases SSNA), as discussed by Carter and Goldstein [18], so it may be reasonably assumed that a larger sample of cognitive-stress responders would have led to expected data.

### Effects of stressors on MSNA and SSNA in the same participants

No differences of statistical significance were found between any two corresponding time points in the eight participants from which we were able to gather both MSNA and SSNA data. It has been previously shown that sympathetic nerve activity does not vary to a great extent within an individual over the course of months to even years [6] in the absence of disease. From this knowledge it may be safe to assume our data were not skewed by the time interval of approximately 1–4 months between visits. Our data suggests that each of the stressors has the same level of impact on both MSNA and SSNA within individuals. That is, an individual will react in the same manner regarding muscle and skin response when subjected to stress.

### Limitations

One of the primary limitations of this study involved the inability to conduct each stress procedure on every participant, reducing the sample sizes for the experiment. Overall, this was not a great issue, with sample sizes reflecting and, in some cases, exceeding prior work; however, this became a more pronounced limitation in the comparison of MSNA and SSNA within participants. Here, the sample size was only eight and further reduced for three of the four stressors. As such, this served to undermine the accuracy of the results for this section of the analysis—especially since the cognitive stressors could not be separated into responders and non-responders. Additionally, with regards to the sample, experiments were conducted on a young, healthy cohort. While this does mean the results presented are representative of the healthy population, they do not reflect stress responses in the elderly or in disease states, which may warrant further investigation. It may also be worth noting that the menstrual cycle of the female participants was not accounted for. There are differing results in the literature as to its effects on sympathetic nerve activity, with some studies reporting no changes during rest and cognitive stressors, and other studies showing profound increases in resting MSNA depending on the stage of the cycle [31, 41]. Nevertheless, there is a possibility it may have impacted our results. Furthermore, our experiments were conducted in a thermoneutral environment (room temperature 22 °C), yet past studies have gone a step further and implemented the use of a customised tube-lined suit to maintain neutral skin temperature [12, 36]. Experimenting in such a way would perhaps account for some of the discrepancies in our SSNA results.

## Conclusions

Our results have shown physical stressor effects on MSNA and SSNA primarily consistent with the literature. From this, a baseline could be established to verify the efficacy of PSTH as an analysis tool—one that could be applied to compare MSNA and SSNA. This was done as such in participants from whom both types of sympathetic activity were recorded. The data showed that stress responses do not vary between MSNA and SSNA on an individual basis. In further applying PSTH analysis to the cognitive stressors, the present study has provided support for the contentious notion that cognitive stress tasks are capable of increasing MSNA and SSNA. This statement would seem to be more applicable for the mental arithmetic task than the Stroop colour–word conflict test, possibly as a result of the level of perceived stress. However, cognitive stress responses are a subject that requires further investigation, especially with regards to SSNA. Our own hypothesis that the cognitive stressors would be more efficacious at altering SSNA than the physical stressors would seem to be true, yet mental arithmetic elicited the greatest changes in MSNA, despite our initial conjectures. Standardisation of the facts surrounding sympathetic responses to various stress conditions is needed to advance the field.

## Data Availability

The data are available upon reasonable request.
